# Development and validation of a novel anoikis-related gene signature for predicting prognosis in ovarian cancer

**DOI:** 10.18632/aging.204634

**Published:** 2023-04-05

**Authors:** Shuangfeng Qian, Yidan Wen, Lina Mei, Xiaofu Zhu, Hongtao Zhang, Chunyan Xu

**Affiliations:** 1 Department of Gynaecology and Obstetrics, Huzhou Maternity & Child Health Care Hospital, Huzhou 313000, China; 2 Department of Sterilization and Supply, Tangdu Hospital, Air Force Military Medical University, Xi'an 710032, China; 3 Department of Gastroenterology, Huzhou Maternity & Child Health Care Hospital, Huzhou 313000, China; 4 Department of Reproductive Medicine, Huzhou Maternity & Child Health Care Hospital, Huzhou 313000, China; 5 Department of Obstetrics and Gynecology, Sichuan Jinxin Women and Children’s Hospital, Chengdu 610000, China

**Keywords:** anoikis, ovarian cancer, gene signature, prognosis, immune environment

## Abstract

Anoikis plays a critical role in variable cancer types. However, studies that focus on the prognostic values of anoikis-related genes (ANRGs) in OV are scarce. Cohorts with transcriptome data and corresponding clinicopathologic data of OV patients were collected and consolidated from public databases. Multiple bioinformatics approaches were used to screen key genes from 446 anoikis-related genes, including Cox regression analysis, random survival forest analysis, and Kaplan-Meier analysis of best combinations. A five-gene signature was constructed in the discovery cohort (TCGA) and validated in four validation cohorts (GEO). Risk score of the signature stratified patients into high-risk (HRisk) and low-risk (LRisk) subgroups. Patients in the HRisk group were associated with worse OS than those in the LRisk group in both the TCGA cohort (p<0.0001, HR=2.718, 95%CI:1.872-3.947) and the four GEO cohorts (p<0.05). Multivariate Cox regression analyses confirmed that the risk score served as an independent prognostic factor in both cohorts. The signature's predictive capacity was further demonstrated by the nomogram analysis. Pathway enrichment analysis revealed that immunosuppressive and malignant progression-related pathways were enriched in the HRisk group, including TGF-β, WNT and ECM pathways. The LRisk group was characterized by immune-active signaling pathways (interferon-gamma, T cell activation, etc.) and higher proportions of anti-tumor immune cells (NK, M1, etc.) while HRisk patients were associated with higher stromal scores and less TCR richness.

In conclusion, the signature reveals a close relationship between the anoikis and prognosis and may provide a potential therapeutic target for OV patients.

## INTRODUCTION

Ovarian cancer (OC) is one of the most malignant tumors of the female reproductive system [1]. Ovarian cancer was characterized by a high mortality rate because most patients are diagnosed with the advanced-stage cancer [2]. OC is often diagnosed with invasion of peripheral organs and distant metastasis. The standard treatment for this cancer is tumor cytopenia combined with platinum-based chemotherapy [3], which has improved the prognosis a lot. However, chemotherapy resistance and high recurrence rate still exist due to its complex pathogenesis [4]. Therefore, reliable prognostic biomarkers for OC are urgently needed for aiding clinical decision-making and improving prognosis [5]. Further studies are necessary to identify such biomarkers.

Anoikis is a novel form of programmed cell death, which is important for the survival of tumor cells without extracellular matrix (ECM) [6, 7]. It can regulate the dynamic balance of the microenvironment by eliminating dislocated cells upon cell detachment from the extracellular matrix (ECM) [8]. Anoikis plays an essential role in tumor invasion and metastasis [9, 10]. The initiation of anoxia apoptosis resistance can help isolated cells escape the death signal pathway, allowing cells to survive under adverse conditions [11, 12].

Recent studies have shown that apoptosis plays an important role in the malignant progression of tumors, including breast cancer [13], lung cancer [14], pancreatic cancer [10], gastric carcinoma [15], endometrial cancer [16] and ovarian cancer [17]. OC cells were mainly exposed to TGF- β1 to stimulate *SOX2* and inhibit anoikis. Transforming growth factor-β (TGF-β) can also regulate epithelial to stromal transition (EMT) through *SMAD3*, which is related to anoikis resistance [17]. Platelets promote cancer cell proliferation and EMT, inhibit anoikis, enhance cancer cell extravasation, and promote immune escape of tumor cells in circulation [18]. Hypoxia in the tumor environment may drive the activation of the *p38-Hur-SOD2* axis, resulting in a decrease in the sensitivity of the tumor to anoikis [19]. The above studies have shown that apoptosis was associated with tumorigenesis and metastasis of OC. In spite of this, few reports have focus on the prognostic value of the ANRGs for OC patients from the perspective of bioinformatics.

In recent years, ANRGs have been shown to have promising potential as a prognostic biomarker for various forms of cancer, including glioblastoma [20], endometrial carcinoma [21], head and neck squamous cell carcinoma [22]. In this study, a multigene signature of ANRGs has been developed using a comprehensive collection of public data to predict the prognosis of ovarian cancer (OC) patients and to elucidate the potential mechanisms involved.

## MATERIALS AND METHODS

### OC datasets

All transcriptome and clinicopathologic data of OC patients were derived from TCGA and GEO databases, comprising the discovery cohort (TCGA-OV, n = 375) and four validation cohorts (GSE32062, n = 260; GSE19829, n = 28; GSE30161, n = 58; GSE71729, n = 125; GSE57495, n = 63). The RNA-seq data of TCGA dataset were downloaded using the new version of the R package “TCGAbiolink”. The gene expression profiles were quantified as Trans Per Million (TPM) transformed values. Corresponding clinical data including follow-up information were downloaded from the UCSC Xena data portal (https://xenabrowser.net/). Similarly, all transcriptome and clinicopathologic data from GEO cohorts were downloaded from GEO database (https://www.ncbi.nlm.nih.gov/geo/). Expression levels were normalized using the “limma” R package while the “Idmap2” R package was utilized to map probes to gene symbols with multiple probes mapped to a single gene by selecting the largest median value. Furthermore, the gene expression data of all patients were z-score transformed. Supplementary Table 1 provides a detailed overview of the collected information.

### Collection of the anoikis-related genes (ANRGs)

ANRGs were collected from the GeneCard database (https://www.genecards.org/). To ensure the consistency of the analysis, a total of 446 genes were obtained, which exist in each cohort (Supplementary Table 2).

### Development and validation of a robust anoikis-related prognostic signature

ANRGs associated with survival were identified using univariate Cox regression analysis of overall survival (OS) in the TCGA cohort and adjusted *p*-value < 0.05 was necessary. To further screen key genes necessary for prognostic signature development, the “randomForestSRC” R package was utilised to perform random survival forest analysis with 2000 repetitions. Each gene with prognostic significance was sorted by an important factor and 10 genes with the relative largest coefficient were selected to align and perform 1023 signature combinations. Through multivariate Cox regression models operation for each combination, a risk score for each combination was calculated based on the expression of each signature gene and its corresponding regression coefficient. All cases were then divided into high-risk (HRisk) and low-risk (LRisk) subgroups according to the median value of the risk score in each combination. Kaplan Meier (KM) analysis was executed for each condition and the best signature was selected based on the smallest *p*-value [23, 24]. Finally, a prognostic anoikis-related signature was generated in TCGA cohort. It was validated in four GEO cohort. The difference is that the best cut off values of Kaplan–Meier curve were used in GEO cohorts, which were calculated by “survminer” R package. To ensure sufficient power, each group's patient number constituted more than 30% of the total cohort. The risk scores of individual patients were assessed in relation to their clinicopathological information (Table 1).

**Table 1:** The relationship between risk score and clinicopathologic information in TCGA cohort.

	**HRisk**	**LRisk**	**p.overall**
	**N=187**	**N=188**	
Age	61.0 [53.0;71.0]	57.0 [50.0;65.0]	0.002
Stage:			0.667
I/II	10 (5.38%)	13 (6.99%)	
III/IV	176 (94.6%)	173 (93.0%)	
Grade:			0.187
G1/G2	26 (14.3%)	17 (9.29%)	
G3/G4	156 (85.7%)	166 (90.7%)	

### Assessment of the prognostic signature

The prognostic value of the signature was evaluated by performing KM analysis, Cox regression analysis and nomogram construction. The “Survival” R package was employed for KM analysis to all cohorts, while the “stats” R package was used for principal component analysis (PCA) between HRisk and LRisk groups in the TCGA cohort. Furthermore, multivariate Cox regression was carried out to test its independently predictive power in all cohorts. Finally, a predictive nomogram was constructed by incorporating the independent predictive factors identified in the TCGA cohort. Its predictive capacity was then investigated by decision curve analysis (DCA) and corresponding calibration analysis.

### Functional enrichment analysis in TCGA cohort

Using the “limma” package for R, we performed differential expression analysis between HRisk and LRisk subgroups. Genes with absolute log2 fold-change > 0.5 and false discovery rate (FDR) < 0.05 were identified as differentially expressed genes (DEGs). Subsequently, DEGs were annotated by enrichment analysis of Gene Ontology biological process (GO-BP) using R package “clusterProfiler” [25]. FDR < 0.05 was established as statistically significant. With the same R package, Gene Set Enrichment Analysis (GSEA) analysis of Kyoto Encyclopedia of Genes and Genomes (KEGG) pathways was performed, wherein FDR < 0.25 was determined to be statistically significant. To assign activity estimates of 50 HALLMARK pathways to each tumor sample, the “GSVA” R package [26] was used to quantify scores, followed by differential analysis through the “limma” R package.

### Relationship between risk score and tumor immune microenvironment (TIME)

In this study, we selected cellular estimates [27], 22 immune cells of Cibersort [28] and TCR richness [29] as TIME-relevant molecular signatures. “IOBR” R package [30] was utilized to quantify the scores of all TIME-relevant molecular signatures.

### Single-cell RNA sequencing (scRNA-seq) data

GEO database [31] was accessed to acquire scRNA-seq profiles (GSE154600) and clinical information [31]. Seurat R package (version 4) [32] was applied to conduct the scRNA-seq analysis. Standard of the quality control was set to “nFeature_RNA >200”, “nCount_RNA<20000” and “percent.mt < 10”. And then 43,057 cells were generated for downstream analysis. “NormalizeData” function was applied to normalized data and “FindVariableFeatures” function was used to identify 2000 highly variable genes (HVGs). After scaling the data, PCA was carried out to reduce dimension based on the HVGs. According to the performance of ElbowPlot, the top 10 PCs were selected for clustering analyses. Subsequently, FindClusters function (resolution = 0.5) was applied to further split the cells into 26 clusters, with cell type determined by CellMarker database [33] and “SingleR” R package [34]. The landscape of the cell types was visualized through TSNE plots.

### Statistical analysis

All the statistical analyses were performed on R v. 4.1.2. The Wilcoxon test was utilized to compare two groups while the Chi-square tests were employed to examine the associations of categorical variables. The associations of risk score with OS were examined by Kaplan–Meier method and Cox proportional hazard analyses. And the log-rank test was employed. All statistical p-values were two-sided.

## RESULTS

**Figure 1:**
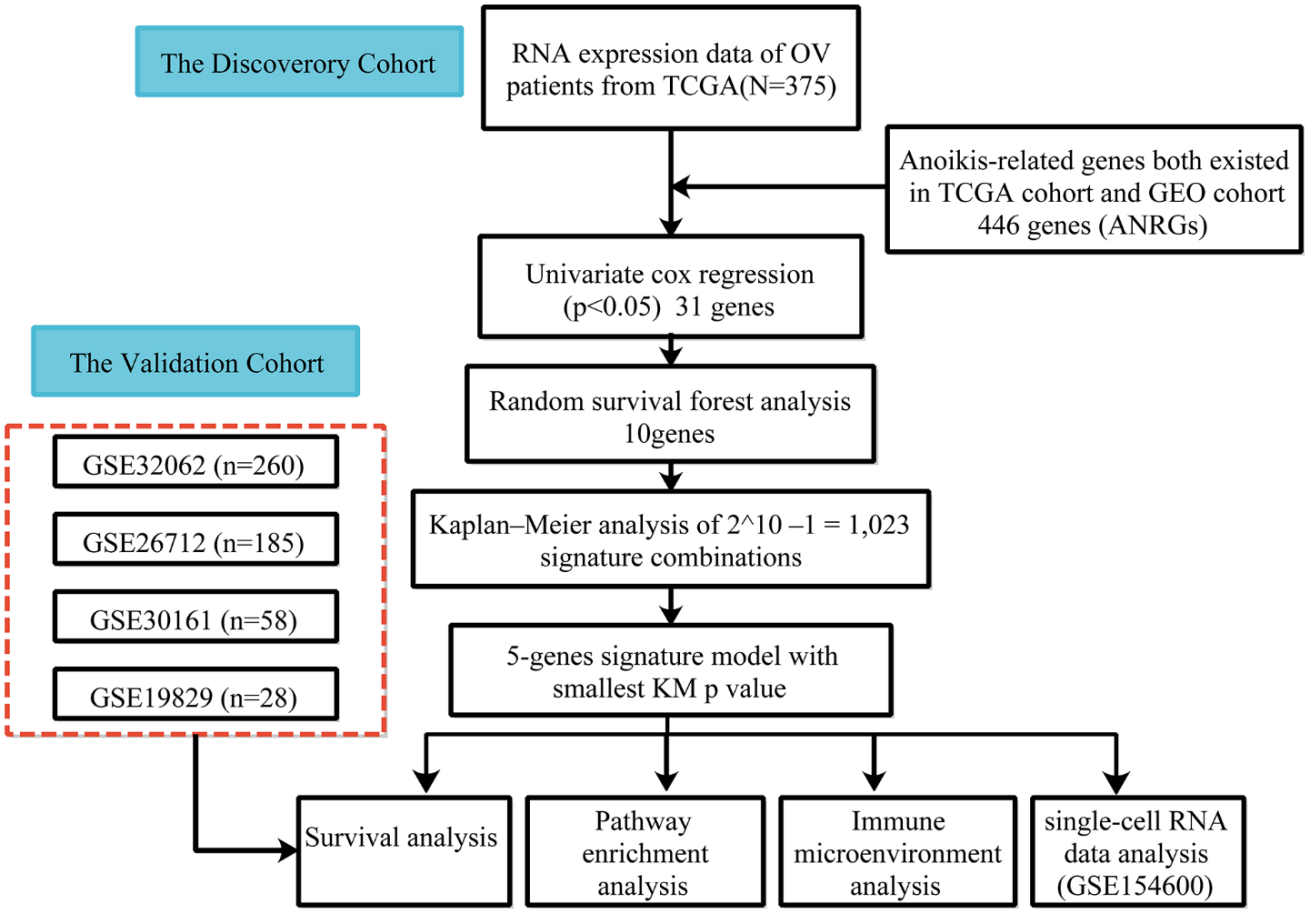
Flow chart of data collection and analysis.

The cohort design and analysis ideas for the entire study were shown in Figure 1. A total of 375 OC patients from the TCGA and 531 OC patients from the GEO were included in this study.

### Construction of an anoikis-related prognostic signature with good performance in the discovery cohort

**Figure 2:**
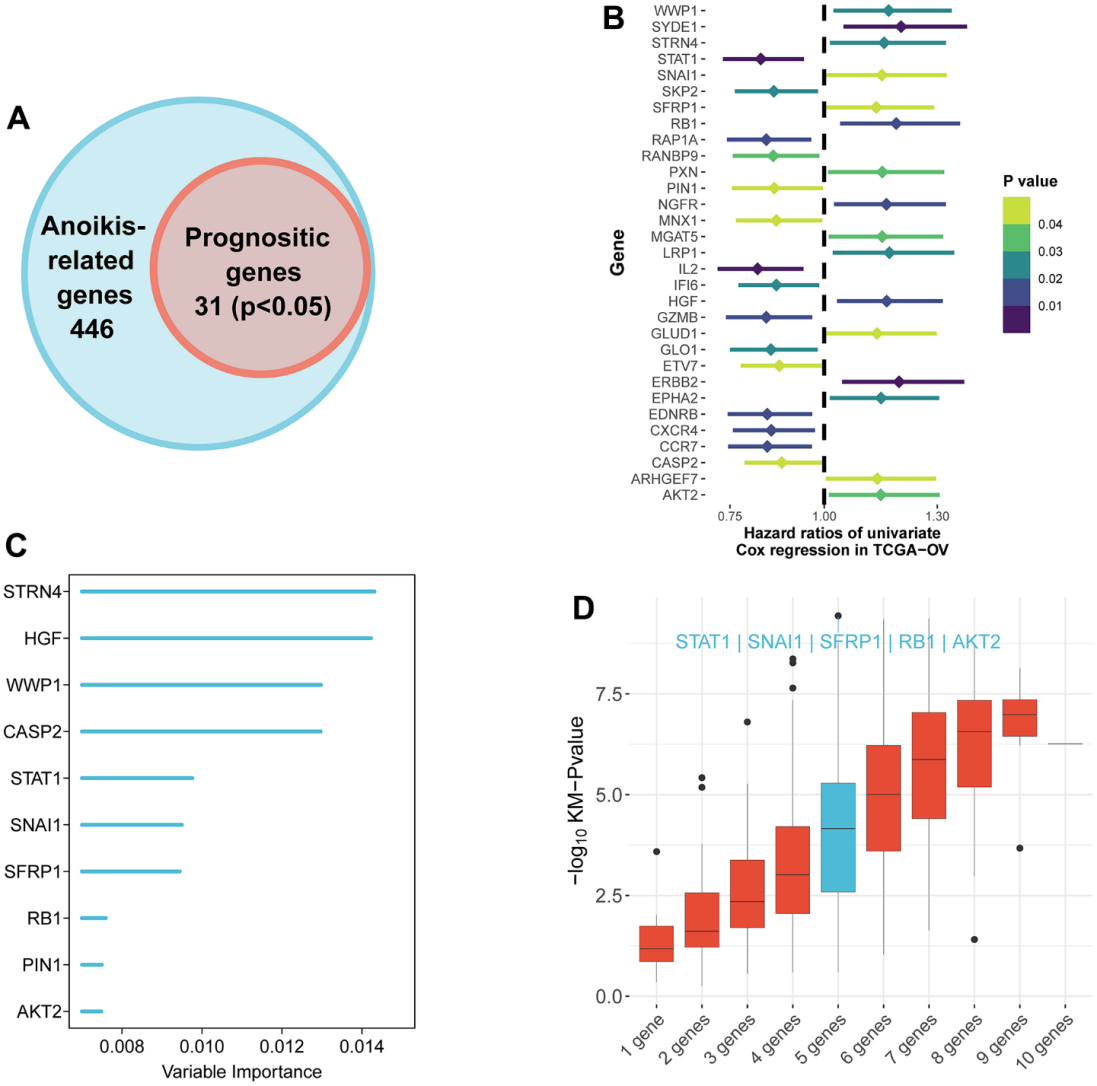
**Identification of the candidate anoikis-related genes in the TCGA cohort.** (**A**) Venn diagram to identify anoikis-related genes that were related to OS. (**B**) Forest plots showing the results of the univariate Cox regression analysis between gene expression and OS. (**C**) Random survival forest analysis screened 10 genes sorted by importance. (**D**) After KM analysis of 2^10 ‒1 = 1,023 combinations, the KM p value of all possible signatures were displayed by boxplots. And the signature included five genes that were screened out, for it had a biggest −log10 *p* value.

**Figure 3:**
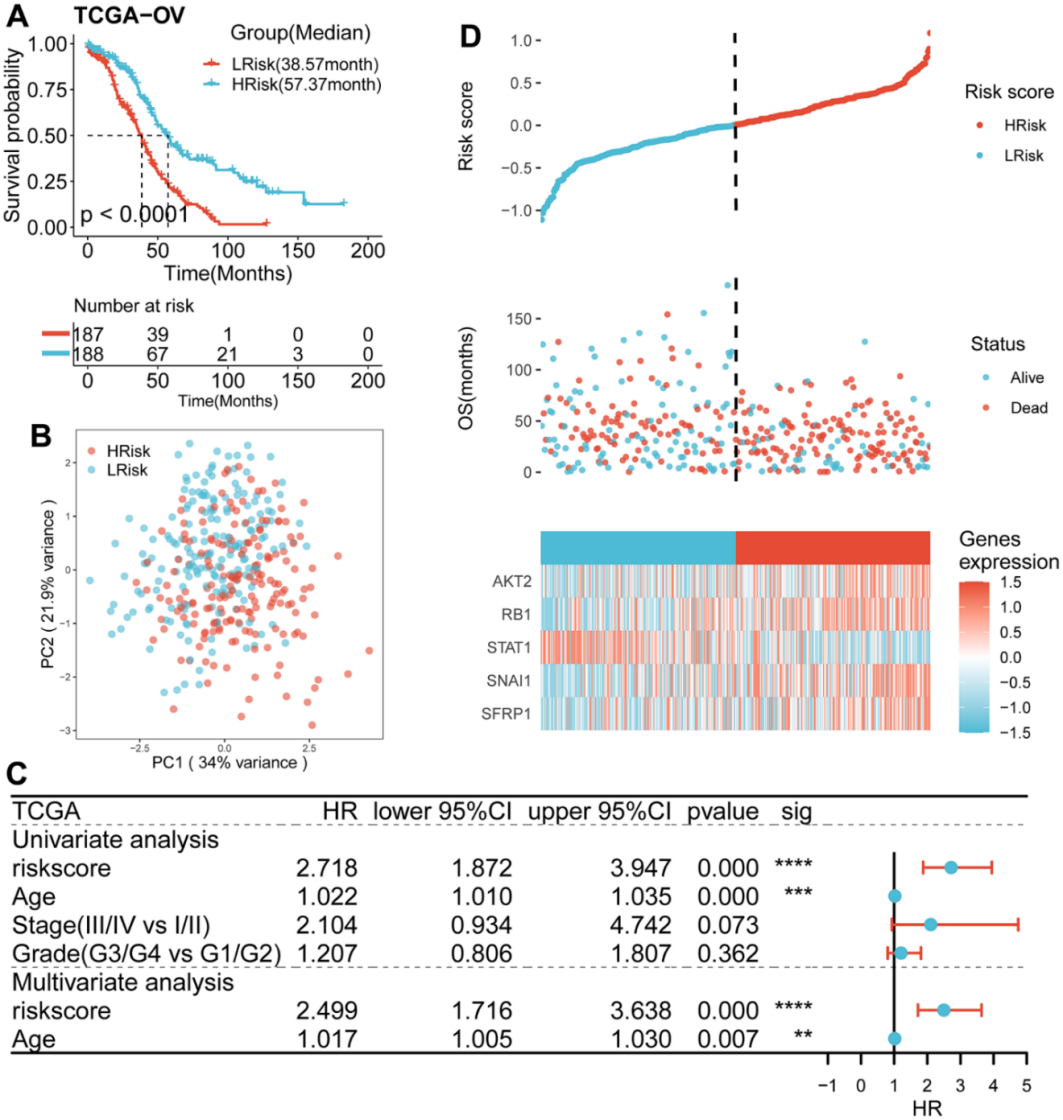
**Survival analysis and prognostic values of the 5-gene signature model in the TCGA discovery cohort.** (**A**) Kaplan-Meier curves for the OS of patients between the HRisk and LRisk group (Log-rank test, *p*<0.001). (**B**) PCA plot of patients in different groups. (**C**) Results of the univariate and multivariate Cox regression analyses regarding OS in the TCGA cohort. (**D**) The distribution of the risk scores. The distributions of OS status, OS and risk score in the TCGA cohort. Heatmap plot for mRNA expression of 5 genes between the HRisk and LRisk group. Expression values were z-score transformed.

**Figure 4:**
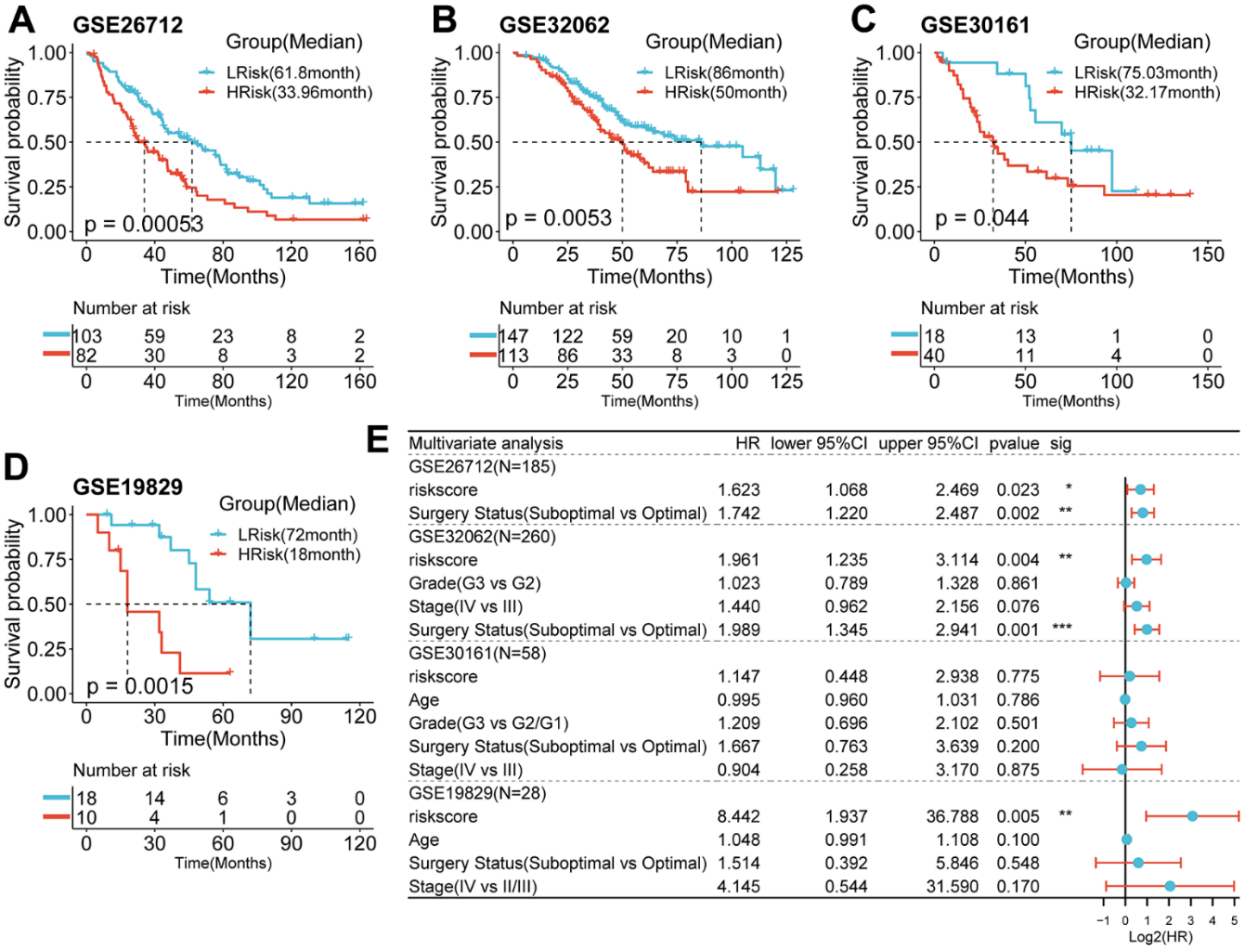
**Survival analysis and prognostic values of the 5-gene signature model in the GEO validation cohorts.** (**A**–**D**) Kaplan-Meier curves for the OS of patients between the HRisk and LRisk group in the GSE26712(A, *p*<0.001), GSE32062(B, *p*=0.005), GSE30161(C, *p*=0.044), GSE19829(D, *p*=001)(Log-rank test). (**E**) Results of the univariate and multivariate Cox regression analyses regarding OS in the GEO cohort.

According to univariate Cox regression analysis, a total of 31 genes associated with anoikis had a prognostic effect on OS in TCGA cohort (Figure 2A, 2B). To reduce the number of signature genes and optimize the prognostic model, a randomized survival forest analysis was employed to assign an importance rank to each prognostic ANRGs (Supplementary Table 3). Based on this analysis, the top 10 genes in terms of importance were selected for KM analysis of combinations (2^10^ -1 = 1,023, Figure 2C). Boxplots of p-values were generated under each condition, indicating that a signature of 5 genes (*RB1*, *STAT1*, *SNAI1*, *SFRP1*, and *AKT2*) had the smallest p-value (Figure 2D and Supplementary Table 4). Notably, except the *STAT1*, the other four genes were identified as risk factors for survival prognosis. Consequently, the anoikis-related prognostic signature was constructed by the modulating expression levels of the five genes following the formula: risk score = (0.049*expression of *SFRP1*) + (-0.275*expression of *STAT1*) + (0.145*expression of *SNAI1*) + (0.189*expression of *RB1*) + (0.130*expression of *AKT2*). KM analysis of the TCGA cohort demonstrated that patients in the LRisk group had significantly better OS compared to those in the HRisk group (log-rank test *p*<0.0001, Figure 3A). Furthermore, PCA indicated that patients in different subgroups were divided into different direction (Figure 3B). Higher number of survivors were observed in the LRisk subgroup (Figure 3D). With the exception of *STAT1*, higher levels of *RB1, SNAI1*, *SFRP1*, *AKT2* mRNA expression were found in the HRisk subgroup (Figure 4C).

### The relationship between the prognostic model and clinical information in TCGA cohort

As shown in Table 1, compared with HRisk patients, the low-risk subgroup was meaningfully associated with younger age. However, no differences were detected in terms of stage and grade.

### Performance of the prognostic signature in the validation cohort

Using the same formula and best cut-off value, the predictive capacity of the prognostic model was verified in four GEO cohorts. Statistically significant worse prognosis was detected among high-risk patients compared to low-risk patients in the GSE26712 (Figure 4A, p<0.001), GSE32062 (Figure 4B, p=0.005), GSE30161 (Figure 4C, p=0.044), GSE19829 (D, p=001) cohort (Log-rank test).

### Predictive efficiency of the prognostic signature

**Figure 5:**
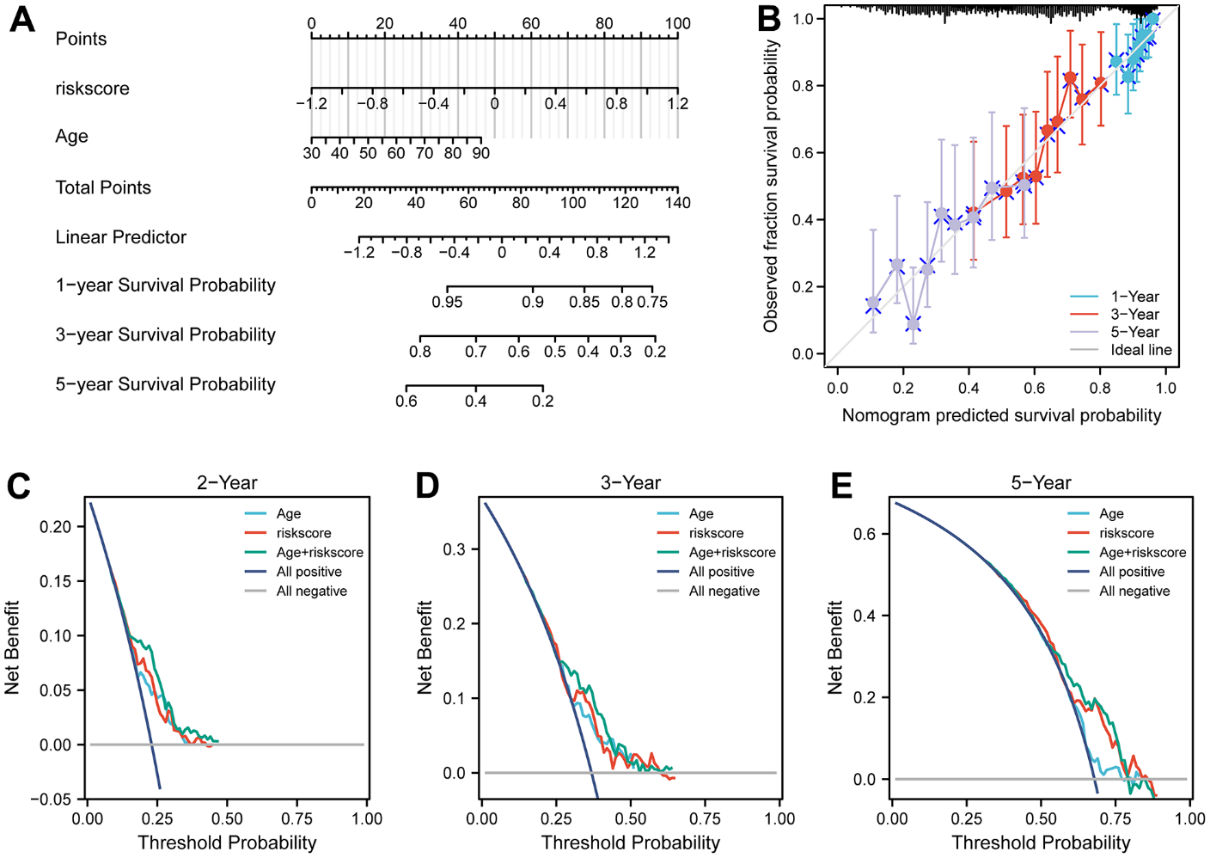
**Nomograms for predicting the probability of patient mortality based on risk score and clinical variables in TCGA cohort.** (**A**) Nomograms plots of TCGA cohort. (**B**) Plots depict calibration of nomograms based on riskscore in terms of agreement between predicted and observed 1-year, 3-year, and 5-year outcomes in TCGA cohorts. Nomogram performance is shown by the plot, relative to the 45-degree line, which represents the ideal prediction. (**C**–**E**) Decision curve analyses of the nomograms based on OS in TCGA cohort for 1-year, 3-year, and 5-year risk.

To examine the independence of the signature, univariate and multivariate Cox analyses were implemented in both cohorts. In the discovery cohort, age and risk score were verified to be independent prognostic indicators (Figure 3C). The same result was found in the validation cohorts except GSE30161, where multivariate Cox analysis revealed that risk score was also an independent factor (Figure 4E). Furthermore, surgery status was also determined to be an independent risk factor in the GSE26712 and GSE32062 cohorts. Furthermore, a predictive nomogram based on age and risk score was established using TCGA data (Figure 5A). The accuracy of this model was judged by the calibration curves between predicted and observed 1-year, 3-year, and 5-year outcomes (Figure 5B). DCA analysis also further highlighted its superior predictive performance (Figure 5C–5E).

### Functional enrichment analysis of the ANRGs-related signature in TCGA cohort

**Figure 6:**
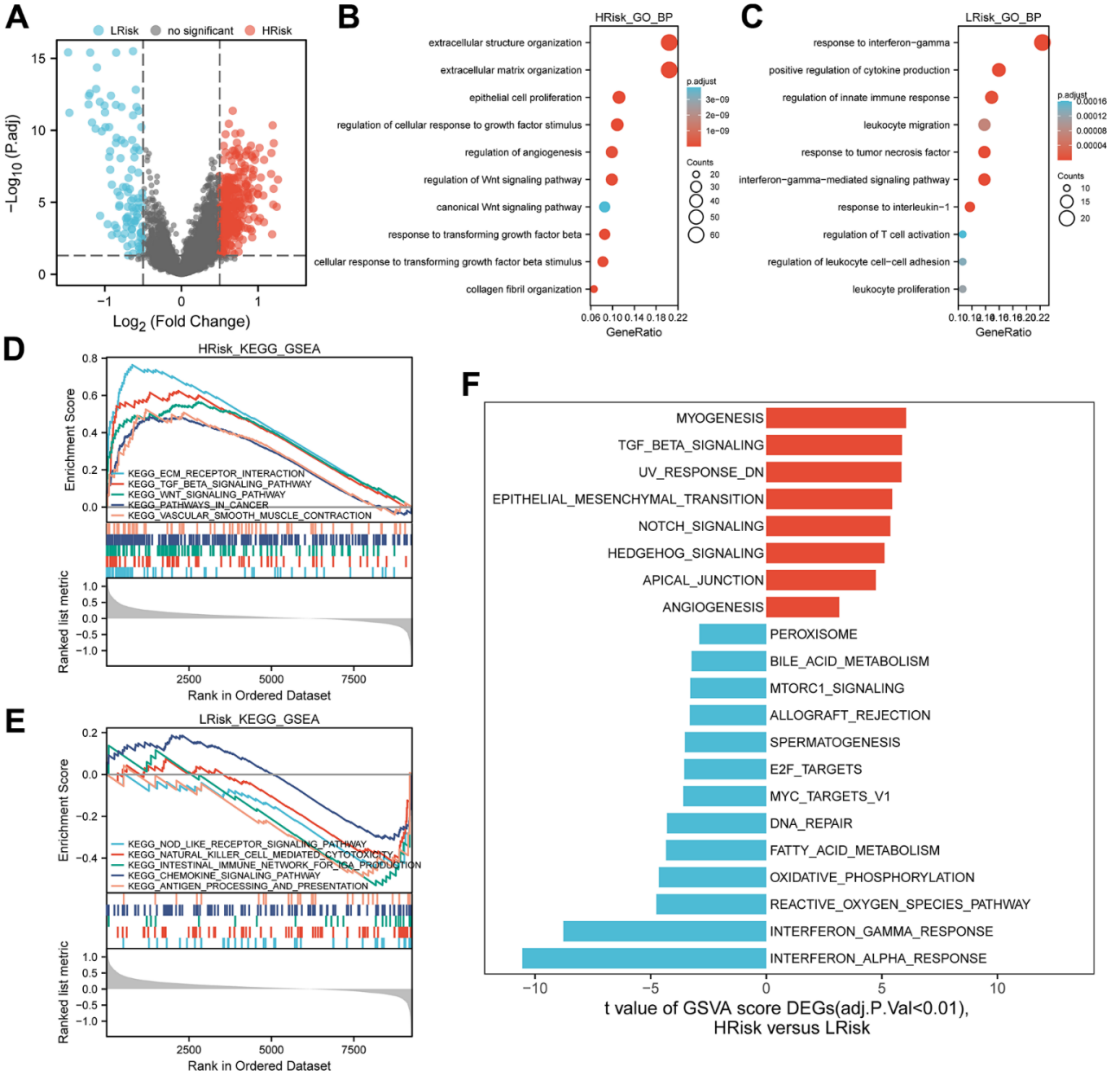
**Representative results of pathway analysis.** (**A**) Volcano plot displayed DEGs between the HRrisk and LRisk subgroups (FDR<0.05, |log2 fold-change|>0.5). (**B**, **C**) Gene Ontology (GO) enrichment analysis of 322 genes up-regulated in the HRisk group (**B**) and 101 genes up-regulated in the LRisk group (**C**). (**D**, **E**) GSEA analysis of KEGG gene sets in the HRisk (**D**) and LRisk (**E**) subgroups (FDR<0.25). (**F**) GSVA analysis of hallmark pathways in the TCGA cohort was performed. Differential analysis of GSVA score between HRisk and LRisk group were displayed and FDR<0.01 were controlled.

After differential expression analysis, 322 DEGs were up-regulated in the HRisk subgroup and 101 DEGs up-regulated in the LRisk subgroup were generated (Figure 6A). GO-BP enrichment analysis of the DEGs, KEGG analysis of all genes and HALLMARK pathways differential analysis indicated that HRisk patients were associated with extracellular matrix (ECM), angiogenesis, transforming growth factor-β (TGF-β) and Wnt/β-catenin pathways (Figure 6B, 6D, 6F), while LRisk patients showed associations with immune-active signaling pathways, including interferon-gamma, T cell activation and immune response (Figure 6C, 6E, 6F).

### Analysis of TIME relevant molecular signatures in TCGA cohort

**Figure 7:**
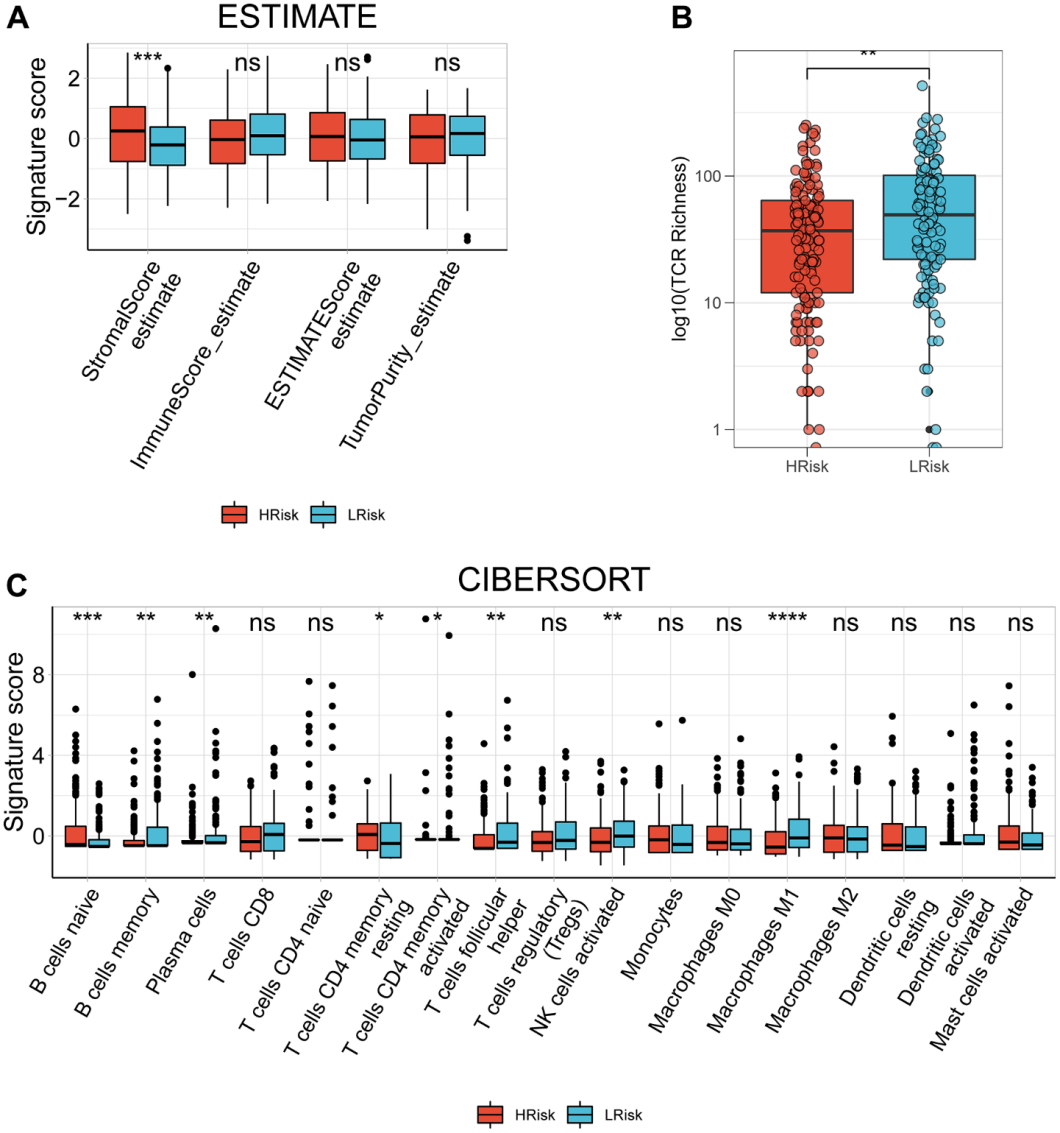
**The correlation between the signature and TIME in TCGA cohort.** (**A**) Comparison of StromalScore, ImmuneScore, ESTIMATEScore and TumorPurity between the HRisk and LRrisk patients. (**B**) Comparison of TCR richness between the HRisk and LRrisk patients. (**C**) Boxplots depicting the CIBERSORT scores of 22 immune cells of the HRisk patients compared to LRrisk patients. (Wilcoxon test, Adjusted P values were showed as: ns, not significant; *, *p*< 0.05; **, *p* < 0.01; ***, *p* < 0.001).

Compared with LRisk patients, HRisk patients got a significantly higher stromal score (Figure 7A). Although the *p*-value was not meaningful, LRisk patients got a higher immune score. Moreover, the TCR richness was found to be significantly higher in the LRisk subgroup (Figure 7B), accompanied by higher abundances of B cells memory, plasma cells, T cells CD4 memory activated, T cells follicular helper, NK cells activated and Macrophages M1 in this group as compared to the HRisk patients (Figure 7C). On the contrary, HRisk patients exhibited a higher abundance of B cells naïve and T cells CD4 memory resting (Figure 7C).

### ANRGs expression in OC TME-associated cells

**Figure 8:**
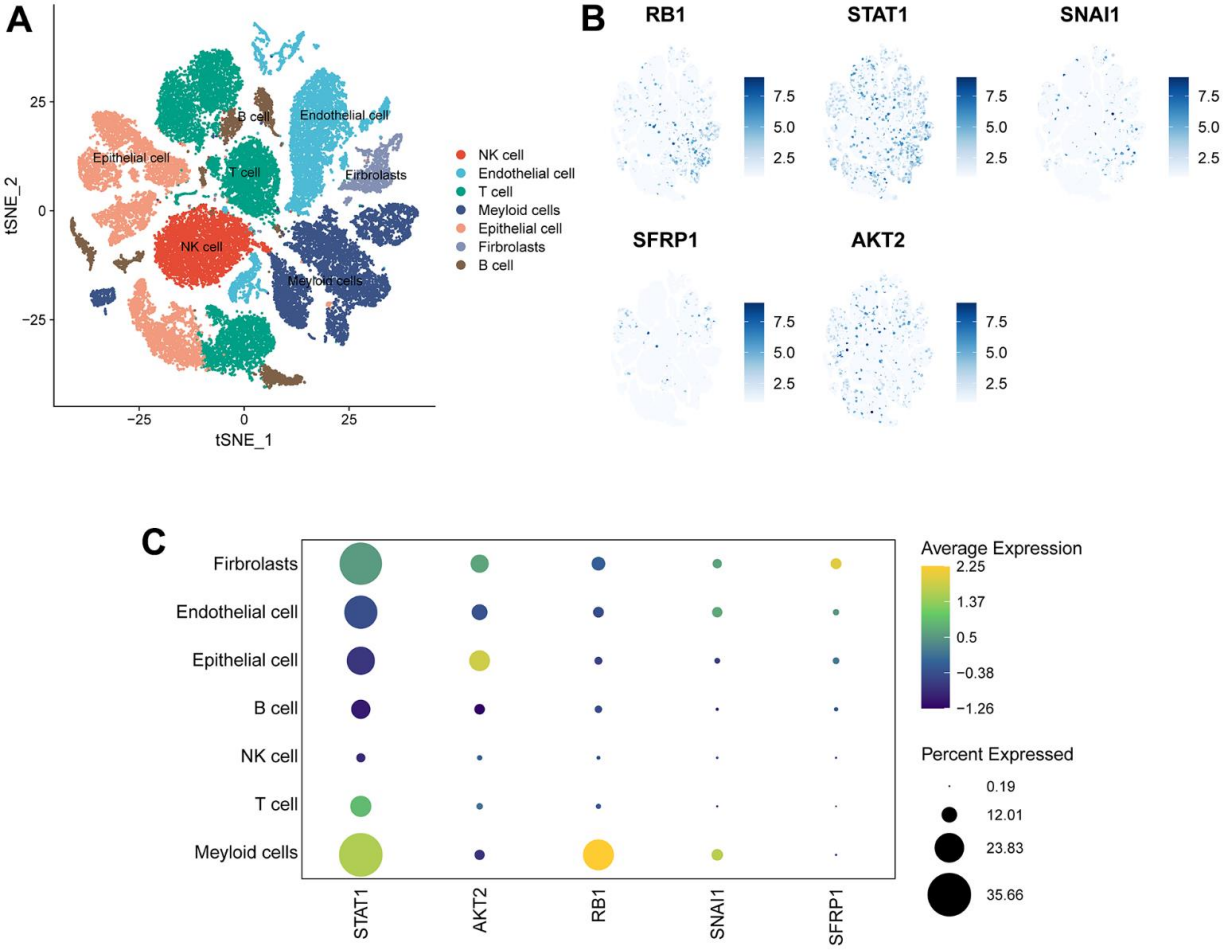
**Expression of 5 signature genes in OV TME-associated cells.** (**A**) TSNE visualization of 43,057 single cells, color-coded by cell type. (**B**) Feature plots depicting the expressions of 5 signature genes (RB1, SNAI1, STAT1, SFRP1, AKT2) in all cell types. (**C**) Dotplot showing the percentages and expressions of 5 signature genes (RB1, SNAI1, STAT1, SFRP1, AKT2) among all cell types.

The scRNA-seq dataset (GES154600) was used to investigate the specific expression of five signature genes in variable TME-associated cells. In the downstream analysis, 7 cell types were annotated in OC TME (Figure 8A). Notably, STAT1 was expressed at high levels in variable cell types, with fibroblasts and myeloid cells exhibiting particularly elevated expression. AKT2 was mainly expressed in malignant epithelial cells and fibroblasts. Expression of SNAI1 and RB1 was generally low with the exception of their transcription in myeloid cells. Lastly, SFRP1 was almost exclusively expressed in fibroblasts (Figure 8B, 8C).

## DISCUSSION

Due to delayed diagnosis and treatment, OC patients were at high risk of a poor prognosis inevitably although more advanced treatment were developed in recent years. Therefore, effective prognostic biomarkers are still urgent requirement for OC patients. The rapid spread of malignancies resulted in aggressive progression of ovarian cancers [35]. Aggressive progression of OCs is often associated with metastasis to the intraperitoneal cavity and peritoneum [36]. Therefore, construction of predictive signature based on genes associated with metastasis may provide a novel and effective strategy for disease management.

A key step in metastasis is the ability of tumor cells to survive under non-adhesion conditions and to escape anchoring-dependent cell death, called anoikis. This process is related to the increased ability of tumor cells to scavenge the increased reactive oxygen species caused by detachment [19]. The intrinsic pathways (DNA damage and endoplasmic reticulum stress) and the extrinsic pathways (mitochondria) were both associated with anoikis [37].

Essentially, anoikis is one of the important steps to prevent transferring tumor cells. Inhibition of anoikis in TME may contributed to tumor migration and metastasis [38, 39]. Anoikis resistance was found to be caused by multiple complex pathways [9, 40–42]. Targeting the ANRGs may provide a novel approach to overcome metastasis of OC. Hence, further investigations into the potential correlation between ANRGs and prognosis in OC patients are warranted.

This study was the first attempt at establishing a prognostic model based on comprehensive ANRGs in OC. We created the signature with stringent criteria and credible algorithm. The prognostic signature established in the discovery cohort included 5 ANRGs (*RB1*, *STAT1*, *SNAI1, SFRP1*, and *AKT2*), all of which have been reported to be associated with anoikis. The prerequisite for the mutation of *Rb1* family to cause tumor in nude mice is to maintain the contact between cells and cells so as to prevent cells from anoikis [43]. Inactivation of the *RB1* pathway resulted in the activation of mTOR pathway and resistance to anoikis [44]. Interferon-γ activated the STAT1 pathway, leading to a decrease in the psoriatic proteins induced by anoikis [45]. *SNAI1* exerts its essential role in colorectal tumor progression and metastasis by regulating EMT pathway [46]. By mediating the activation of Wnt pathway, the down-regulation of *SFRP1* promoted breast epithelial cell proliferation and resistance to anoikis [47]. Furthermore, the absence of mtDNA promotes migration to basement membrane proteins by activating *AKT2* and downstream products of prostate epithelial cells to promote resistance to anoikis [48]. It has also been reported that *AKT2* ablation stimulated PC-3 cell migration in terms of prostate cancer [49]. These five key genes have been identified to be closely related to anoikis; however, there are relatively few studies on ovarian cancer. Future exploration of the underlying mechanisms of ovarian cancer is thus critical.

To explore the potential mechanism of the signature, functional enrichment analysis was applied. The results indicated that HRisk patients were associated with pathways known to promote tumor progression, such as extracellular matrix (ECM), angiogenesis, TGF-β and WNT signaling pathways. All these signaling have been reported to promote the development of OC. For example, down-regulation of *PAX8* gene expression has been reported to reduce the ability of tumor cells to migrate and adhere to the ECM, thereby reducing anoikis resistance [50]. Molecular characterisation of angiogenesis-related genes was associated with OC heterogeneity and offers new therapeutic approaches [51]. OC cells were mainly exposed to TGF- β1 to stimulate SOX2 and inhibit anoikis [17]. Furthermore, down-regulation of β-Catenin has been shown to inhibited tumor growth and peritoneal metastasis in OC tumor model by stopping the formation of OC spheroids, which protected tumor cells from anoikis [52]. In contrast, LRisk subgroup was associated with antitumor-related pathways, primarily those related to immune activation, such as interferon-gamma, T cell activation and immune response. Immunosuppression is known to promote tumor progression, and the activation of anti-tumor immunity may explain the better prognosis of LRisk patients.

In recent years, TME have been identified as a new hotpot of research for OC. Different immune cells play different roles in the development of ovarian cancer. Cox proportional hazards model shows that highly infiltrated CD8+ TILs are associated with shorter disease-specific survival and overall survival [53].

It has been shown that M1 macrophage exhibits anti-tumor effects, whereas M2 pro-tumor effects [54]. This unique property of NK cells and their ability to enhance T cell responses and antibodies supports NK cells as a therapeutic hope in the fight against cancer, as they have the potential to kill tumor cells in a variety of ways without prior sensitisation [55]. The above mentioned tumor-killing immune cells are highly infiltrated in the low-risk group. OC patients with shorter survival had a higher infiltration of B cells naïve and T cells CD4 memory resting [56], which existed in the HRisk group. This pointed out patients with high-risk score were considered to be comprise immune escape factors and tumor-promoting factors.

Despite the promising results from the bioinformatics analysis in OC, this study still has certain limitations. Firstly, the unavailability of some clinical information hindered us from fully exploring the relationship between the model and clinical features and developing a better nomogram, such as the details of treatment, pathological details of the tumor. Secondly, although we used multiple cohorts in this study to validate the reliability of the model, an external cohort was necessary for verifying. Moreover, experimental analyses of *in vitro* and *in vivo* in real-world is the most effective way to explore the mechanism of signature genes.

## CONCLUSIONS

In conclusion, a novel and robust prognostic signature of ANRGs in OC was constructed. This model was demonstrated to be independently predictive factor for survival in both the discovery (TCGA) and validation (GEO) cohorts, providing appropriate patient stratification and treatment guidance for future trials. The potential mechanism of the ANRGs-related signature was explored in terms of pathway and TIME analysis. However, it is not enough, and we need further exploration of our study.

## Supplementary Material

Supplementary Table 1

Supplementary Table 2

Supplementary Table 3

Supplementary Table 4
