# Supplementary material for: Development and validation of a novel anoikis-related gene signature for predicting prognosis in ovarian cancer
Source: Aging (Albany NY). 2023 Apr 5;15(9):3410–26. doi: 10.18632/aging.204634 (PMC10449303; doi:10.18632/aging.204634)
Supplement: Supplementary Table 1 [file aging-15-204634-s001.pdf]

SUPPLEMENTARY TABLE

Supplementary Table 1. Collecting datasets of ovarian cancer with survival data.

Datasets	Platform	Number of samples	Information	Ref(PMID)
TCGA-OV	HGU133A	375	OS	21720365
GSE32062	GPL6480	260	OS	22241791
GSE19829	HGU133 Plus 2.0|GPL570	28	OS	20547991
GSE30161	HGU133 Plus 2.0|GPL570	58	OS	22348014
GSE26712	HGU133A|GPL96	185	OS	18593951
