# Supplementary material for: Development and validation of a novel anoikis-related gene signature for predicting prognosis in ovarian cancer
Source: Aging (Albany NY). 2023 Apr 5;15(9):3410–26. doi: 10.18632/aging.204634 (PMC10449303; doi:10.18632/aging.204634)
Supplement: Supplementary Table 3 [file aging-15-204634-s003.pdf]

**Supplementary Table 3. Importance of each ANRGs with prognostic value.**

| Surv.rf.importance | Symbol |
|---|---|
| 0.000249198422485996 | EPHA2 |
| 0.00130624196315903 | IFI6 |
| 0.00155186347980523 | CXCR4 |
| 0.00179512513650021 | ETV7 |
| 0.00221351532216709 | IL2 |
| 0.00253653812525469 | EDNRB |
| 0.00273673948984161 | GLUD1 |
| 0.00293103931042791 | MGAT5 |
| 0.00317421179400052 | PXN |
| 0.00317906854041056 | GLO1 |
| 0.00396332522758073 | GZMB |
| 0.00418758898717 | RAP1A |
| 0.00448999451495502 | SYDE1 |
| 0.00467633071727487 | LRP1 |
| 0.00476357098300726 | NGFR |
| 0.0051250211506144 | MNX1 |
| 0.0051452601040497 | CCR7 |
| 0.0053291884206191 | ARHGEF7 |
| 0.00575030911675733 | ERBB2 |
| 0.00629400305287395 | RANBP9 |
| 0.0072773616628405 | SKP2 |
| 0.0074811901848788 | AKT2 |
| 0.00749574568838223 | PIN1 |
| 0.00759282638541233 | RB1 |
| 0.00944359874109739 | SFRP1 |
| 0.00949671396884303 | SNAI1 |
| 0.00976025270965955 | STAT1 |
| 0.0129669044286207 | CASP2 |
| 0.0129751446640335 | WWP1 |
| 0.0142266388169153 | HGF |
| 0.01431475729316 | STRN4 |
